# Mechanisms of Intron Loss and Gain in the Fission Yeast *Schizosaccharomyces*

**DOI:** 10.1371/journal.pone.0061683

**Published:** 2013-04-17

**Authors:** Tao Zhu, Deng-Ke Niu

**Affiliations:** MOE Key Laboratory for Biodiversity Science and Ecological Engineering and Beijing Key Laboratory of Gene Resource and Molecular Development, College of Life Sciences, Beijing Normal University, Beijing, China; University College London, United Kingdom

## Abstract

The fission yeast, *Schizosaccharomyces pombe*, is an important model species with a low intron density. Previous studies showed extensive intron losses during its evolution. To test the models of intron loss and gain in fission yeasts, we conducted a comparative genomic analysis in four *Schizosaccharomyces* species. Both intronization and de-intronization were observed, although both were at a low frequency. A de-intronization event was caused by a degenerative mutation in the branch site. Four cases of imprecise intron losses were identified, indicating that genomic deletion is not a negligible mechanism of intron loss. Most intron losses were precise deletions of introns, and were significantly biased to the 3′ sides of genes. Adjacent introns tended to be lost simultaneously. These observations indicated that the main force shaping the exon-intron structures of fission yeasts was precise intron losses mediated by reverse transcriptase. We found two cases of intron gains caused by tandem genomic duplication, but failed to identify the mechanisms for the majority of the intron gain events observed. In addition, we found that intron-lost and intron-gained genes had certain similar features, such as similar Gene Ontology categories and expression levels.

## Introduction

Spliceosomal intron densities vary greatly among different organisms [1]–[4]. Although losses and gains of introns in evolution have been confirmed by numerous studies, their mechanisms have not been fully revealed [5]–[12].

Three models of intron loss have been proposed: the reverse transcription (RT) model, the genomic deletion model, and the model of non-homologous end joining (NHEJ) repair of double strand breaks [7]–[9]. In recent years, many attempts have been made to test these models by phylogenetic analysis of the presence and absence of introns among orthologous genes. Among the three models, the RT model has been widely tested. It was initially proposed to explain the 5′-biased distribution of the limited number of introns in the budding yeast *Saccharomyces cerevisiae* [13]. In this model, the 3′ side of a mature mRNA is more successfully reverse-transcribed because of the occasional dissociation of reverse transcriptase from template mRNAs. Recombination of the partial cDNA products with genomic DNA causes exact loss of an intron or introns from the gene. Its first prediction is that introns are preferentially lost from the 3′ sides of genes. This pattern has been observed in *Dictyostelium discoideum*, *Schizosaccharomyces pombe*, *Mycosphaerella*, *Cryptococcus*, *Caenorhabditis elegans*, *Anopheles*, and mammals [14]–[19], but not in *Fusarium graminearum*, *Magnaporthe grisea* or rice [20], [21]. In *Neurospora crassa* and *Aspergillus*, the biased position of intron loss has been observed in a comparative analysis among distantly related species [18], but not in analyses of closely related species [20], [22]. In both *Arabidopsis* and *Drosophila*, conflicting results have been reported [10], [23]–[26]. A modified version of the RT model is that reverse transcription is primed by the polyA tail itself and, therefore, the preferential loss of which introns depends on the specific secondary structure of the mRNA molecules [27], [28]. It could explain the intron losses that were not biased to the 3′ sides of genes. However, this modified version has not received further support [10], [22]. The second prediction of the RT model is that adjacent introns tend to be lost simultaneously. This prediction has been confirmed in *Cryptococcus*, *Fusarium*, *Aspergillus*, *Drosophila* and mammals [10], [15], [16], [19], [22], but not in *Caenorhabditis*, *Plasmodium* or *Arabidopsis* [23], [29], [30]. In addition, homologous recombination between cDNA and genomic DNA would produce an exact intron loss. By contrast, in the genomic deletion model, introns are lost individually and often imprecisely by unequal exchange of alleles. In most previous studies, especially those focused on distantly related species, filtration of unreliable alignments artificially excluded all possible cases of imprecise intron loss. Up to now, only a few cases of imprecise intron loss have been detected in pufferfish, *Drosophila*, and Muridae [10], [31], [32]. Recently, it was proposed that introns could be lost either precisely or imprecisely during the NHEJ repair of double strand DNA breaks [9]. By analyzing the frequency of micro-homology between splice sites of lost introns, researchers observed evidence for the NHEJ model in *Arabidopsis* [23], but not in *Drosophila* [10]. This micro-homology would degrade gradually during evolution; therefore, this signal could only be detected in closely related organisms.

Compared with intron loss, the possible mechanisms underlying intron gain are much more diverse. At least six mechanisms have been proposed for intron gain: intron transposition, transposon insertion, group II intron insertion, tandem genomic duplication, intron transfer and NHEJ-mediated intron gain events [11], [33]. The NHEJ-mediated intron gain, which is similar to the NHEJ model of intron loss, has been frequently observed in *Daphnia* and *Aspergillus* [22], [34]. The intron transposition model suggests that an intron is reverse-spliced into a different position of its own mRNA or another mRNA. Subsequently, recombination of the cDNA reverse-transcribed from the mRNA with genomic DNA would create a new intron [33]. Therefore, the newly gained intron would have high sequence similarity with an intron in the same gene or an unrelated gene. Evidence for this model has been found in *Oikopleura* and *Mycosphaerella* [14], [35]. Insertions of transposable elements containing splicing signals may create new introns. The test of this model requires similarity between gained introns and transposons. A few such cases have been found in *Drosophila* and *Arabidopsis* [10], [23]. In *Cladosporium* and *Dothistroma*, introner-like elements (ILEs) were found to contribute to new introns [36], [37]. However, further study is required to show whether the ILEs were inserted similarly to transposons or via reverse splicing. New introns may also arise among tandem genomic repeats containing cryptic splice sites. This tandem genomic duplication model has also gained some support [19], [38]. Finally, introns may be transferred between paralogs or genes with highly similar segments by gene conversion. A few cases of intron gain by this model have been observed in *Chironomus*, *Aspergillus* and *Mycosphaerella* [14], [22], [39]. Studies on intron gain were extensively reviewed in [11].

A broad definition of intron loss and gain could include de-intronization and intronization, which are conversions of intron sequences to exon sequences, and vice versa, by mutations [40]–[42]. Intronization and/or de-intronization events have been found in *Cryptococcus*, *Fusarium*, *Caenorhabditis*, mammals and *Populus* [15], [40], [41], [43], [44]. However, detection of intronization/de-intronization events depends heavily on the quality of gene annotation. Caution must still be taken when relevant transcriptome data are limited [22].

**Figure 1:**
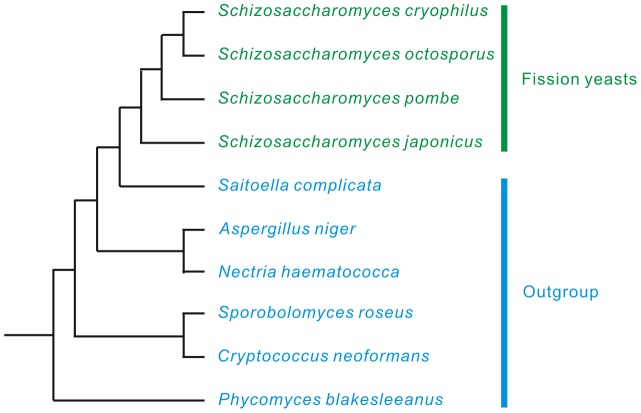
Phylogenetic relationship of the fission yeasts and six outgroup fungal species. The tree was constructed from a study on comparative genomics of fission yeasts [47] and the NCBI Taxonomy database (http://www.ncbi.nlm.nih.gov/taxonomy). It is not scaled according to phylogenetic distances.

The fission yeast, *Schizosaccharomyces pombe*, has a low intron density [2], [45]. Its high quality gene annotations and high coverage of transcriptomes make it a good candidate for the study of intron evolution. Previous studies indicated that it has experienced extensive intron losses during evolution [17], [18], [46],[47]. However, all the studies were comparisons of *S. pombe* with distantly related organisms, such as vertebrates and plants. Most of the evolutionary traces of intron loss and gain could not be retained over such a long evolutionary history. For this reason, we compared four closely related species; *Schizosaccharomyces cryophilus*, *Schizosaccharomyces octosporus*, *S. pombe* and *Schizosaccharomyces japonicus* with six outgroup fungus species (Figure 1). Certain novel findings obtained in *Schizosaccharomyces* enriched our understanding of the mechanisms of intron evolution.

## Materials and Methods

### Genomes and Gene Annotations

We downloaded the genome sequences and gene annotations of four fission yeast species (*S. cryophilus*, *S. octosporus*, *S. pombe* and *S. japonicus*) from the Broad Institute (http://www.broadinstitute.org/science/data, September 08, 2012). Data for *Saitoella complicata*, *Aspergillus niger*, *Nectria haematococca*, *Sporobolomyces roseus*, *Cryptococcus neoformans* and *Phycomyces blakesleeanus* were obtained from JGI (http://genome.jgi.doe.gov/, September 08, 2012). *Saitoella complicata* was selected because it is closely related to the *Schizosaccharomyces* genus and the other five outgroup species were selected because of their relative abundance of introns. The phylogenetic relationship between the *Schizosaccharomyces* species and the outgroup species is shown in Figure 1.

Genes with obvious annotation errors, such as those having coding sequences with non-multiples of three nucleotides or those conflicting with their protein sequences, were discarded. If a gene had multiple transcript isoforms, the longest mRNA was retained for analysis. As alternative splicing events in fungi are rare compared with plants and animals [47], [48], this selection, even if inaccurate, would not affect our final results significantly.

### Detection of Orthologs

First, the best reciprocal BLAST was used to search for orthologous protein-coding genes across the four *Schizosaccharomyces* taxa, with thresholds of *E*<10^−10^ and identity ≥0.25. Then, all the possible orthologs were imported into OrthoCluster 2.0 [49] to generate synteny blocks among the four organisms. The minimum orthologous gene number and the maximum mismatched gene number in each block were set to 3. Only orthologous gene pairs that were located in synteny blocks were retained to avoid retrogenes or processed pseudogenes being mistaken as true orthologs. In total, 2,963 1∶1∶1∶1 orthologous genes were found, among which 2,108 intron-containing groups were used for further analysis.

### Identification of Unique Intron Positions

**Figure 2:**
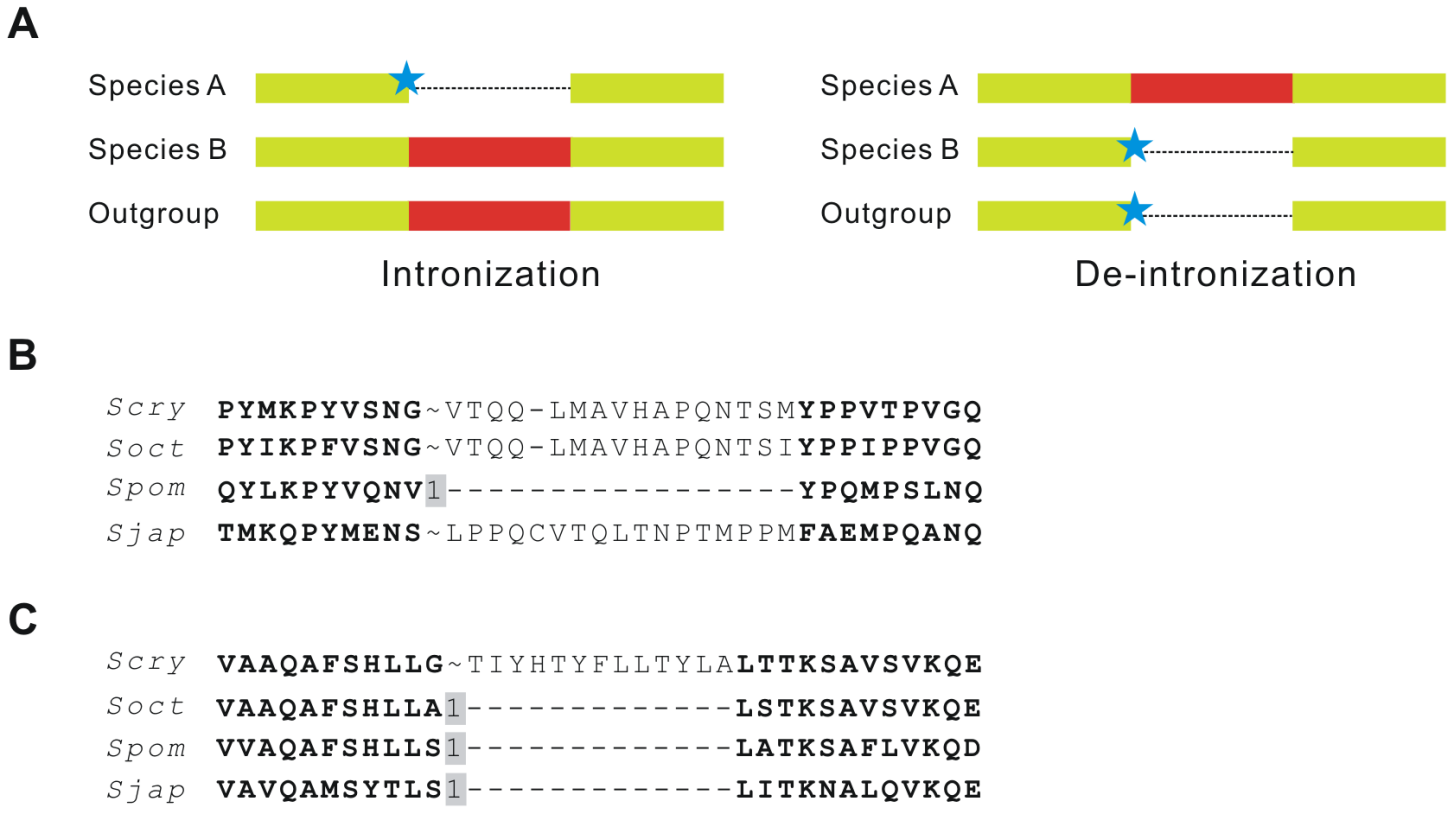
Detection of intronization and de-intronization. Both intronization and de-intronization are characterized by introns neighboring large gaps while the surrounding coding regions remain well aligned. These two can be distinguished by the presence or absence of the introns in other outgroup species (A). The conserved surrounding coding regions are marked in yellow and the exonized or intronized regions are marked in red. Introns are represented as stars. The protein alignments show a case of intronization in *S. pombe* (B) and a case of de-intronization in *S. cryophilus* (C). Intron phases are marked as 0, 1, 2 or ∼ (absence of an intron). Species names abbreviations: *S. cryophilus* (*Scry*), *S. octosporus* (*Soct*), *S. pombe* (*Spom*), and *S. japonicus* (*Sjap*).

Each group of orthologous proteins was aligned using MUSCLE 3.8 [50] and intron positions were mapped onto the alignments. Position candidates for intron loss and gain were filtered using the following criteria: a) Adjacent intron positions in different taxa that were less than five amino acids in distance were excluded as they might represent intron sliding events; b) Introns near large gaps (longer than five amino acids), which might represent possible intronization or de-intronization events, were manually checked and analyzed separately (Figure 2); c) If the identity of 15 amino acids neighboring an intron position on each side was less than 0.30, the first quartile of all orthologous protein sequence identities, it was discarded because of poor alignment. This resulted in 1,775 conserved intron positions (Table S1) and 808 unique intron positions.

Six outgroup fungus species were used to distinguish intron losses and intron gains among the unique intron positions. First, the outgroup orthologous proteins were identified and aligned with the intron-containing *Schizosaccharomyces* proteins using the same method mentioned above. Second, for each candidate intron position, the presence and absence of introns in each species were marked as 0 (lacks an intron), 1 (has an intron) or ? (no orthologous regions). This data list was imported into the Dollop (Dollo and Polymorphism Parsimony) program in the PHYLIP 3.6 [51] package to detect intron loss and gain events that happened in the taxa and nodes within the *Schizosaccharomyces* group. Finally, considering the limitation of Dollop (Text S1) and the empirically low occurrence of intron gains, we identified an intron gain event only when the identification was supported by ≥4 outgroup branches.

### Certification of Target Intron Positions Using Transcriptome Data

In order to exclude the possibility that the unique intron positions, including intron losses/gains and intronizations/de-intronizations, were gene structure annotation artifacts instead of real intron changes, it is necessary to use transcriptome data to identify related gene structures. The RNAseq/Inchworm/PASA assembly sequences of the four fission yeast species were downloaded from the Broad Institute (http://www.broadinstitute.org/annotation/genome/schizosaccharomyces_group/MultiDownloads.html, October 02, 2012). The transcripts were mapped onto the corresponding genomic sequences using BLAT 34 [52]. A target intron position supported by ≥1 RNA assembly was regarded as a transcript-certified intron position. If a target position lacked related transcripts, we searched the related gene in the Feature Search page of Broad Institute (http://www.broadinstitute.org/annotation/genome/schizosaccharomyces_group/FeatureSearch.html) to get additional transcripts that are only available on the webpages.

## Results and Discussion

Among the 2,108 intron-containing orthologs across the four *Schizosaccharomyces* species, we found 1,775 conserved intron positions and 808 unique intron positions. By consulting the orthologous genes in the six outgroup fungal species, we identified 677 putative cases of intron loss and 62 putative cases of intron gain (Tables S2–S3), as well as 156 putative cases of intronization and de-intronization.

### Intronization and De-intronization Are Rare in Fission Yeasts

**Figure 3:**
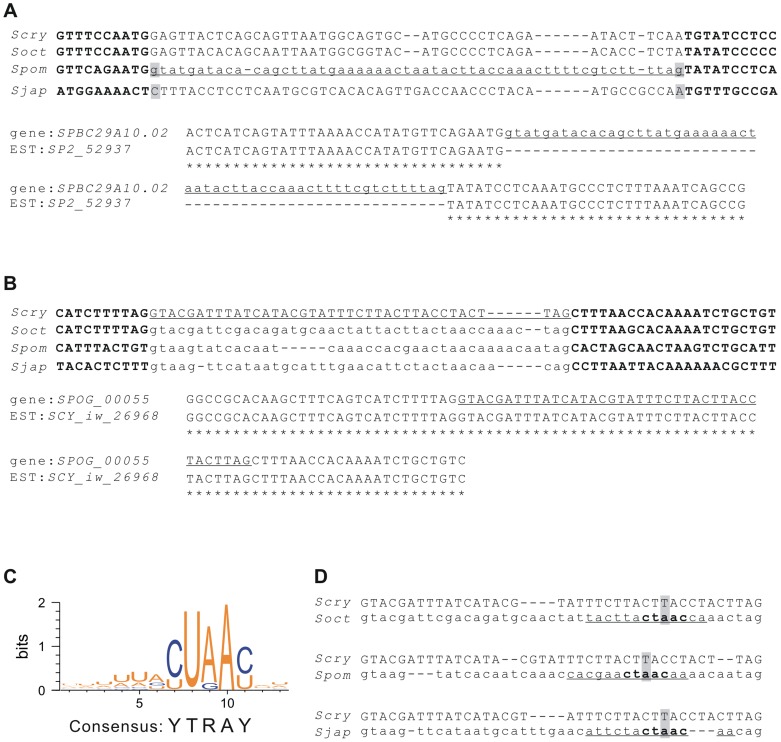
Intronization and de-intronization events in fission yeasts. A) Intronization occurred in the *SPBC29A10.02* gene of *S. pombe*. The intronized region is marked by underlining and variations in splice sites are marked in gray. Alignment of gene *SPBC29A10.02* with its related EST is shown below. B) De-intronization occurred in the *SPOG_00055* gene of *S. cryophilus*. Alignment of *SPOG_00055* with its orthologs shows a de-intronization event, with the exonized region marked by underlining. Alignment of gene *SPOG_00055* with its related EST is shown below. C) The consensus sequence (YTRAY) of branch sites in fission yeasts. Branch site sequences were detected using ICAT [53] and consensus sequences were generated using Weblogo [54]. D) The degraded branch sites of *SPOG_00055* compared with its orthologous intron regions. The branch sites predicted by ICAT are marked by underlining and the consensus regions in the branch sites are in bold. Mutations are marked in gray. The introns are shown in lower case while exonic sequences are presented in upper case. Species name abbreviations: *S. cryophilus* (*Scry*), *S. octosporus* (*Soct*), *S. pombe* (*Spom*), and *S. japonicus* (*Sjap*).

Intronization or de-intronization events are characterized by unique introns neighboring large gaps of exons where the other exon parts remain well aligned (Figure 2). In this study, 156 putative cases of intronization and de-intronization events were detected. When transcriptome data were not available, changes of splicing signals might be used as evidence of intronization and de-intronization. Among the 156 possible cases, we found that changes of splicing signals occurred in 63 cases. By contrast, only two cases were supported by transcriptome data: one case of intronization and one case of de-intronization (Table 1, Figure 3). Surprisingly, changes in splicing signals were observed in the intronization case, but not in the de-intronization case. In addition to these two cases, there were 12 putative cases of intronization/de-intronization that were neither supported nor disproved by transcriptome data. Most of the cases, even those having changes of splicing signals, were disproved by transcriptome data (Table 1). A conclusion can be drawn here is that the identification of intronization and de-intronization depends heavily on the accuracy of genome annotation. The putative intronizations and de-intronizations in some previous studies, such as those in *Aspergillus* [22], are likely to be mostly annotation errors.

**Table 1:** Number of putative intronization and de-intronization events in fission yeasts.

	Intronization	De-intronization	Undetermined^a^
**With changes in splicing signals**			
Supported by transcriptome data^b^	1	0	0
Disproved by transcriptome data^b^	50	1	2
Lacking related transcript data^b^	8	0	1
**Without any change in splicing signals**			
Supported by transcriptome data^b^	0	1	0
Disproved by transcriptome data^b^	77	5	7
Lacking related transcript data^b^	3	0	0

a Unable to distinguish between intronization and de-intronization because of a lack of outgroup evidence.

b For intronization, “Supported by transcriptome data” means that the novel intron region is spliced in at least one transcript. “Disproved by transcriptome data” means that the novel intron region is retained in all the related transcripts. For de-intronization, “Supported by transcriptome data” means the novel exon region is retained in at least one transcript. “Disproved by transcriptome data” means the novel exon region is spliced in all transcripts and is thus an unannotated intron. If no transcripts cover the novel intron or exon region, it is defined as “Lacking related transcript data”.

### Mutations in Splice Sites and Branch Sites Lead to Intronization and De-intronization

In gene *SPBC29A10.02* of *S. pombe*, an intronization event occurred by the conversion of a coding segment into a new intron. At the two ends of this segment, two point mutations (C to G and A to G) created the two splicing sites (Figure 3A).

In the case of de-intronization, the fourth intron of gene *SPBC29A10.14* was conserved among *S. octosporus*, *S. pombe*, and *S. japonicus*. However, the orthologous sequence of the intron had changed into an exonic segment in *S. cryophilus* (Figure 3B). No mutations were detected in 5′ or 3′ splice sites. Using the ICAT program and Weblogo 3.3 [53], [54], we identified the consensus sequence (YTRAY) of intron branch sites in *Schizosaccharomyces* species (Figure 3C). Furthermore, we found an A to T mutation in the branch site of gene *SPOG_00055* in *S. cryophilus* (Figure 3D). This point mutation probably caused the de-intronization by inactivating the branch site.

Multiple signals are required for efficient splicing [55]; therefore, conversion of an exonic segment into an intron requires multiple constructive mutations. The point mutations we observed in gene *SPBC29A10.02* merely represent the end steps. By contrast, mutations in any of the essential signals of splicing (e.g., the 5′ and 3′ splicing signals, the branch sites, and the exonic splicing enhancers) could produce alternative products [55] or even cause de-intronization. Unless cryptic splicing signals are very common, de-intronization is expected to have a higher frequency than intronization. However, de-intronization introduces an insertion and possibly premature stop codons into mRNA, which might be deleterious and be selected against. Therefore, the observed frequency of de-intronization should be much lower than the actual frequency. Previous studies observed a higher frequency of intronization than de-intronization [40], [41].

### Most Intron Loss and Gain Positions Were Supported by Transcriptome Data

The identification of intron loss or gain events were initially based on the gene structure annotations of the four fission yeast species. However, some of these annotations might be erroneous, which could be seen from the fact that most putative intronization or de-intronization events were annotation errors. For intron losses and gains, gene annotation errors could also lead to false-positive results. If an exonic segment was mis-annotated as an intron in orthologous genes, a simple deletion of it would lead to a false-positive case of intron loss. Similarly, a simple insertion of exonic sequence mis-annotated as an intron would lead to a false-positive case of intron gain. Therefore, the transcriptome data were also required to support the target intron loss or gain events.

In our datasets, we found that most intron loss and gain positions were supported by transcriptome data (Tables S2–S3). In only three out of the 677 putative cases of intron loss, the extant orthologous introns were lacking related transcriptome data. Therefore we are not sure whether they are really introns. Among the intron-absent genes, 14 were not covered by any transcripts. These genes might be inactivated after losing their introns or be uncovered simply because of low-coverage transcriptomes. For accuracy, we excluded these 17 cases and thus retained 660 cases of intron loss in the further analyses (Table 2). On the other hand, all 62 putative cases of intron gain were found to be successfully spliced out from pre-mRNAs (Table 2). In contrast with intronization or de-intronization events, most of the intron loss and gain events were supported by transcriptomes.

**Table 2:** The number and rate of intron loss and gain in fission yeasts.

Species name^a^	Extant intron number^b^	Divergence time^c^	Lost introns^d^	Loss rate^e^	Gained introns^d^
*S. cryophilus*	2,263	32	0	0	1
*S. octosporus*	2,242	32	10	1.4×10^−10^	1
Ancestor of *Scry* and *Soct*	2,240	87	85	4.4×10^−10^	13
*S. pombe*	2,134	119	173	6.8×10^−10^	4
Ancestor of *Scry*, *Soct*, and *Spom*	2,003	102	143	7.0×10^−10^	24
*S. japonicus*	2,179	221	249	5.2×10^−10^	19

a Species name abbreviations: *S. cryophilus* (*Scry*), *S. octosporus* (*Soct*), *S. pombe* (*Spom*).

b Only introns that were not filtered out by our criteria were counted, including conserved introns and unique introns. Therefore, they are smaller than the total numbers of annotated introns.

c The divergence time between each species and the nodes were obtained from [47]. Data are shown in millions of years.

d Introns lost or gained at the ancestor nodes were counted only once.

e The rates of intron loss are shown as number of lost introns per year per intron.

Based on the divergence time between taxa and the number of considered introns in each species and nodes, the rate of intron loss was calculated (Table 2). We found that the variation of intron densities within the *Schizosaccharomyces* group was negatively correlated with the intron loss rates (Figure S1). It seems that intron loss was the main force that shaped the gene structures in fission yeasts.

### Evidence for the Genomic Deletion Model of Intron Loss

**Figure 4:**
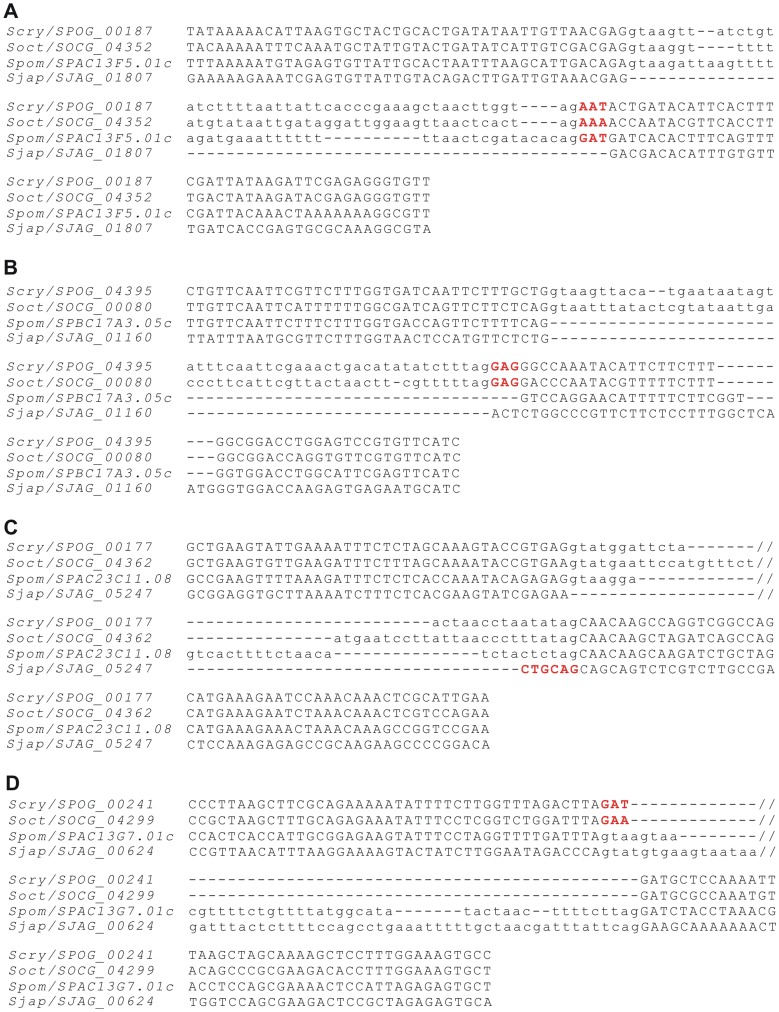
Cases of imprecise intron deletion in fission yeasts. The alignments of DNA sequences around imprecise intron deletion regions are shown. Exon sequences are shown in upper case while intron sequences are shown in lower case. Exonic sequence indels accompanying intron loss are marked in red. Internal regions in long intron sequences are marked by “//”. Species name abbreviations: *S. cryophilus* (*Scry*), *S. octosporus* (*Soct*), *S. pombe* (*Spom*), and *S. japonicus* (*Sjap*).

Although the genomic deletion model of intron loss is widely cited [7], [8], very few supporting cases have been revealed [10], [31], [32]. In our dataset of intron loss, most cases are exact losses of entire introns. Fortunately, we detected four cases of imprecise intron deletions (Figure 4). In the first case, gene *SJAG_01807* lost an intron together with 3 nt of the downstream exon (Figure 4A). In the second case, loss of an intron from gene *SPBC17A3.05c* was also accompanied by loss of 3 nt from the downstream exon. Meanwhile, we found that one of its orthologous genes, *SJAG_01160*, also lost the same intron, but this was a precise loss (Figure 4B). In the third case, an imprecise intron loss occurred in gene *SJAG_05247* when 6 nt of the intron were left on the downstream exon (Figure 4C). In the last case, the orthologous gene pair *SPOG_00241* and *SOCG_04299* both lost an intron at the same position, but left 3 nt of the intron on the upstream exon (Figure 4D). We hypothesize that this represents one intron loss event that occurred in the common ancestor of *S. cryophilus* and *S. octosporus*.

None of these imprecise intron losses caused any frameshifts in the coding sequences. It is very likely a result of negative selection. Imprecise intron losses that caused frameshifts might also have occurred, but have been eliminated. In addition, some cases of imprecise intron losses that did not cause frameshifts might also have been eliminated because of indels of amino acid sequences, especially when the indels were very long. Genomic deletion may also produce exact intron loss (at a low frequency); therefore, we suggest that the genomic deletions that have actually occurred in evolution should be more frequent than the imprecise intron deletions we observed.

In addition, the frequency of genomic deletion might also been underestimated for methodological reasons. In previous studies [10], [31], [32] and the present one, imprecise intron losses all involved leaving or moving very short exon sequences. All the studies focused on intron sites in well-aligned sequence regions; therefore, deletions of large regions would definitely be filtered out and the remaining cases observed might reflect only part of the genomic deletion events that have actually occurred. For this reason, we searched all the remaining unique intron sites that were discarded because of low alignment identity. Two possible cases were found (Figure S2). Unfortunately, we could not find orthologous genes in the outgroup species, and thus failed to distinguish between intron loss and intron gain for these two cases. Further evidence is required to determine whether they represent imprecise intron losses or imprecise intron gains, an unknown phenomenon with no previous reports.

### Intron Loss Events Are Mainly Caused by Reverse Transcription

**Figure 5:**
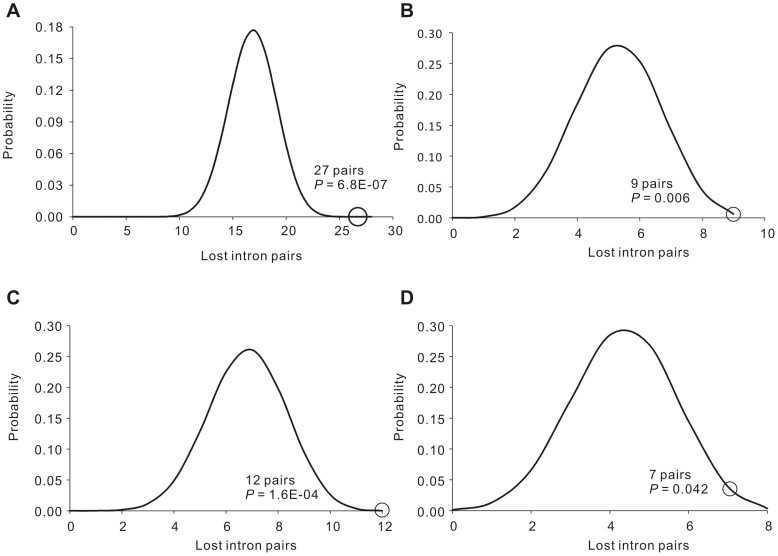
Adjacent introns tend to be lost together in fission yeasts. The probability distribution of all possible numbers of adjacent lost intron pairs is shown, with the observed pattern marked by a circle. The probabilities exceeding the observed numbers of lost intron pairs were small and, therefore, adjacent introns tend to be lost together more frequently than by chance. Lost introns are categorized by A) *S. pombe*, B) *S. japonicus*, C) Ancestor of *S. cryophilus*, *S. octosporus* and *S. pombe*, D) Ancestor of *S. cryophilus* and *S. octosporus*.

Similarly to previous studies in other taxa [15], [16], [19], three predictions of the classical RT model of intron loss have been confirmed in fission yeasts. First, most cases (656 among 660) of the intron losses are precise intron deletions. Second, adjacent introns tend to be lost simultaneously. We observed 38 groups of losses of adjacent introns: six in the ancestor of *S. cryophilus* and *S. octosporus*, 17 in *S. pombe*, seven in the ancestor of *S. cryophilus*, *S. octosporus* and *S. pombe* and eight in *S. japonicus*. Referring to the method of Roy and Gilbert [17], we calculated the probability distribution of the loss of adjacent introns with the assumption of independent loss of each intron (Figure 5). The probabilities that exceed the number of observed lost intron pairs were low enough (0.043∼6.8×10^−7^) to deny the null hypothesis. Furthermore, we observed a preferential loss of introns at the 3′ side of genes. As shown in Table 3, the 3′-biased intron loss is significant in every species and node of the yeasts studied.

**Table 3:** Comparison of the relative positions between lost introns and conserved introns^a^.

	Lost introns		Conserved introns		
	*n*	median	*n*	median	*P*-value
**Grouped comparison** b					
Ancestor of *Scry* and *Soct*	170	0.429	3,550	0.202	9.4×10^−11^
*S. pombe*	173	0.485	1,775	0.204	5.6×10^−16^
Ancestor of *Scry*, *Soct*, and *Spom*	429	0.493	5,325	0.202	3.3×10^−32^
*S. japonicus*	249	0.509	1,775	0.204	8.1×10^−18^
**Paired comparison** c					
Ancestor of *Scry* and *Soct*	64	0.676	64	0.170	7.6×10^−16^
*S. pombe*	52	0.543	52	0.200	0.001
Ancestor of *Scry*, *Soct*, and *Spom*	156	0.590	156	0.169	6.5×10^−12^
*S. japonicus*	90	0.553	90	0.200	1.7×10^−8^

a The relative position was defined as intron position divided by the length of the coding sequence from the 5′ end. For introns lost at the ancestor nodes, the relative position was calculated in each of the species. Species name abbreviations: *S. cryophilus* (*Scry*), *S. octosporus* (*Soct*), *S. pombe* (*Spom*).

b All the relative intron positions were grouped by lost introns and conserved introns and a Mann-Whitney *U* test was used to calculate the *P* values.

c For each intron-lost gene, the median positions of lost introns and conserved introns were paired and a Wilcoxon signed rank test was used to calculate the *P* values. Intron-lost genes with no conserved introns were not counted; therefore, the sample sizes were smaller than grouped comparisons.

Meanwhile, we also tested the NHEJ-mediated model of intron loss by surveying the micro-homology between 5′ and 3′ splice sites of lost introns [9]. The frequency of direct repeats around lost introns was not significantly higher than that around conserved introns (*P*>0.10 in all species and nodes). Thus, the NHEJ-mediated model was not supported by the intron losses in fission yeast.

### Evidence for Tandem Genomic Duplications Leading to Intron Gain

**Figure 6:**
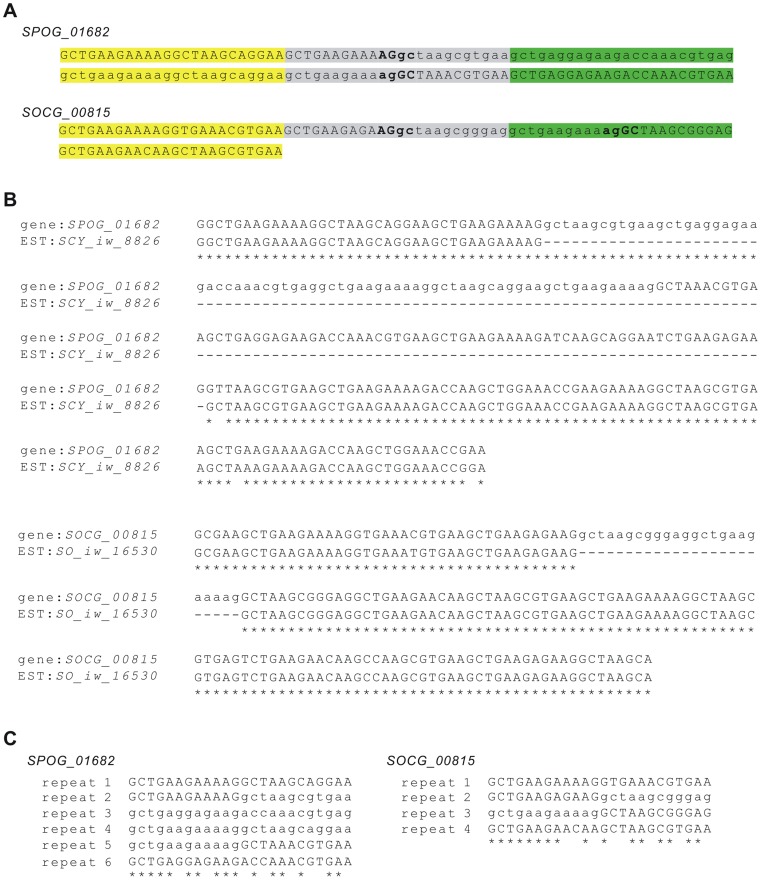
Intron gain caused by tandem genomic duplication in fission yeasts. A) Gained introns and surrounding exon sequences. To show each tandem repeat unit clearly, they are shown in different colors. The cryptic splice sites (AGGC) in tandem repeat units are marked in bold. B) Alignment of the intron-gained genes with their supporting ESTs. C) Alignments of the repeat sequences. They are not fully identical. The introns are shown in lower case while exonic sequences are shown in upper case.

Intron gains are generally observed at a much lower frequency than intron losses. Even for the limited number of intron gains, definite source sequences could not be found for most new introns [22], [34]. This delayed the interpretation of the mechanisms of intron gain and raised doubts concerning the reliability of the identified intron gain events [22]. Similarly, we did not find the source sequences of most of the new introns identified in this study. Among the 62 cases of intron gains, definite source sequences were revealed for only two new introns. We found that the similarity can be extended to the neighboring exons. They were both similar to nearby exons and seemed to result from tandem genomic duplications. Gene *SPOG_01682* gained its intron at position 576-0 (after the 576th amino acid, phase 0) and its ortholog *SOCG_00815* gained its intron at another position 612-0. Both of the introns comprised tandem repeats (Figure 6A). *SPOG_01682* had a spliced EST, *SCY_iw_8826*, which spliced out a longer segment than the annotated intron (Figure 6B). Thus, the true *in vivo* situation of this intron requires further investigation. *SOCG_00815* also had a spliced EST, *SO_iw_16530* and it mapped with the intron correctly, although it only covered a small part of the 5′-side exon (Figure 6B). In addition, the 24-nt repeat units around these two gained introns were not totally identical (Figure 6C), which reduces the possibility of them arising through sequence assembly errors. It seemed that the proto splice sites (AGGC) in the repeat units led to the occurrence of new introns.

We also attempted to test the NHEJ model of intron gain by surveying the frequency of direct repeats near intron-exon boundaries [11], [34]. Among the newly gained introns, 29.2% have direct repeats near boundaries. Comparatively, we found that 25.1% of conserved introns also have direct repeats near their boundaries. A Pearson square test showed that the difference is not significant (*P* = 0.338). Therefore, the NHEJ model of intron gain was not supported in fission yeasts.

### Some Genes are More Likely to Lose and Gain Introns

In our dataset, 58 genes lost multiple introns and five gained multiple introns. It seems that intron number variations are not evenly distributed among different genes, but biased to certain special ones. We tested whether intron-lost (IL) genes and intron-gained (IG) genes are clustered in certain special features. BiNGO 2.44 [56] was used to classify and characterize the analyzed genes into Gene Ontology (GO) categories, which were based on the *S. pombe* GO annotation from the Gene Ontology website (http://www.geneontology.org). Compared with the whole gene sets, IL genes were more likely to participate in metabolism, molecular transportation and enzyme activity regulation (Table 4). Interestingly, IG genes were also clustered in similar GO categories, although the sample size was much smaller (Table 4). We also showed that IL genes and IG genes both had significantly higher expression levels than other genes (Table S4). These results implied that intron loss and gain in fission yeast might share some similar mechanisms.

**Table 4:** Overrepresented GO categories in intron-lost and intron-gained genes.

GO description	Number of target genes	Number of all genes	*P*-value^a^
**Intron-lost genes**			
Catalytic activity*	195	1,616	1.1×10^−14^
Hydrolase activity*	85	629	4.1×10^−8^
Metabolic process	275	2,990	1.0×10^−6^
Nucleobase, nucleoside, nucleotide and nucleic acid metabolic process	130	1,187	2.3×10^−6^
Transferase activity	70	545	5.7×10^−6^
Regulation of biological process	116	1,076	2.3×10^−5^
Catabolic process	81	661	3.9×10^−5^
Binding	170	1,746	6.2×10^−5^
Intracellular	395	4,936	1.7×10^−4^
Response to stimulus	80	778	2.6×10^−3^
Protein binding	80	774	2.2×10^−3^
Kinase activity	24	173	3.0×10^−3^
Macromolecule metabolic process	195	2,206	4.3×10^−3^
Nucleus	229	2,655	5.3×10^−3^
Transport*	83	819	3.2×10^−3^
Membrane	88	901	6.9×10^−3^
Ligase activity	22	164	6.7×10^−3^
**Intron-gained genes**			
Catalytic activity*	29	1,616	6.9×10^−4^
Transport*	18	819	1.3×10^−3^
Hydrolase activity*	15	629	1.7×10^−3^
Transcription regulator activity	3	24	1.9×10^−3^

a A hypergeometric test was used and only GO categories with *P* values lower than 0.05 were shown.

* GO categories that were both present in intron-lost and intron-gained genes. The null hypothesis is that the intron-lost and intron-gained genes were randomly clustered among the GO categories. However, three out of four GO categories of intron-gained genes were shared by intron-lost genes, which implied that intron loss and gain in fission yeast might share some similar mechanisms.

As discussed above, intron losses were mainly mediated by reverse transcriptase. In the current models of intron gain, only the intron transposition model shares a similar mechanism (i.e. a requirement for reverse transcription) with intron loss. However, the intron transposition model requires similarities between newly gained introns and other extant introns, which was not observed in our study. As the selective constraint is much lower for introns than for coding regions, accumulated mutations in gained intron sequences might have resulted in them being significantly divergent from the source sequences. It is also possible that some of the identified intron gains might be intron losses in other taxa, considering the high intron loss rates in fission yeasts. False-positive results of intron gains would cause the IG genes share some false-positive similarities with IL genes.

## Conclusions

In fission yeasts, we found a higher frequency of intron loss than intron gain. Although a moderate number of putative intronization and de-intronization events were observed, most cases were filtered out using transcriptome data. Careful examination of the confident cases of intronization and de-intronization revealed them to be caused by mutations in splice sites and branch sites. Although at a low frequency, imprecise intron deletions were observed, supporting the genomic deletion model of intron loss. The characteristics of most intron losses are consistent with the RT model. Similar to previous studies [22], [34], the source sequences of most newly gained introns identified in this study were not found. Some of the source sequences might have been lost during evolution, or became significantly dissimilar. It is also possible that some of the intron gains might actually be intron losses in other species. In spite of this, evidence for the tandem genomic duplication model was supported by two cases of intron gains. We also found that intron loss rates are not uniform. Some genes, like those participating in metabolism, molecular transportation and enzyme activity regulation, are more likely to lose their introns.

## Supporting Information

Table S1 **Conserved introns across the four fission yeast species.**
(XLS)

Table S2 **Lost introns across the four fission yeast species.**
(XLS)

Table S3 **Gained introns across the four fission yeast species.**
(XLS)

Table S4 **Intron-lost genes and intron-gained genes in fission yeasts have higher expression levels.**
(DOC)

Figure S1 **Number of extant introns and intron loss rate are negatively correlated.** The intron loss rate and the number of extant introns are negatively correlated. *S. cryophilus* has the largest number of extant introns and has experienced the lowest intron loss rate, while *S. pombe* has the fewest introns and had the highest intron loss rate. Species name abbreviations: *S. cryophilus* (*Scry*), *S. octosporus* (*Soct*), *S. pombe* (*Spom*), and *S. japonicus* (*Sjap*).
(TIF)

Figure S2 **Large indels neighboring unique intron positions in fission yeasts.** The alignments of DNA sequences around unique intron regions are shown. Exon sequences are shown in upper case while intron sequences are shown in lower case. Exonic sequence indels accompanying intron loss are marked in red. Species name abbreviations: *S. cryophilus* (*Scry*), *S. octosporus* (*Soct*), *S. pombe* (*Spom*), and *S. japonicus* (*Sjap*).
(TIF)

Text S1 **The limitation of the Dollop program.**
(DOC)

## References

[1] Jeffares DC, Mourier T, Penny D (2006) The biology of intron gain and loss. Trends Genet 22: 16–22. 16290250 10.1016/j.tig.2005.10.006

[2] Mourier T, Jeffares DC (2003) Eukaryotic intron loss. Science 300: 1393. 12775832 10.1126/science.1080559

[3] Rogozin I, Carmel L, Csuros M, Koonin E (2012) Origin and evolution of spliceosomal introns. Biol Direct 7: 11. 22507701 10.1186/1745-6150-7-11 PMC3488318

[4] Jeffares DC, Penkett CJ, Bahler J (2008) Rapidly regulated genes are intron poor. Trends Genet 24: 375–378. 18586348 10.1016/j.tig.2008.05.006

[5] Carmel L, Rogozin IB, Wolf YI, Koonin EV (2007) Patterns of intron gain and conservation in eukaryotic genes. BMC Evol Biol 7: 192. 17935625 10.1186/1471-2148-7-192 PMC2151770

[6] Carmel L, Wolf YI, Rogozin IB, Koonin EV (2007) Three distinct modes of intron dynamics in the evolution of eukaryotes. Genome Res 17: 1034–1044. 17495008 10.1101/gr.6438607 PMC1899114

[7] Rodriguez-Trelles F, Tarro R, Ayala FJ (2006) Origins and evolution of spliceosomal introns. Annu Rev Genet 40: 47–76. 17094737 10.1146/annurev.genet.40.110405.090625

[8] Roy SW, Gilbert W (2006) The evolution of spliceosomal introns: patterns, puzzles and progress. Nat Rev Genet 7: 211–221. 16485020 10.1038/nrg1807

[9] Farlow A, Meduri E, Schlotterer C (2011) DNA double-strand break repair and the evolution of intron density. Trends Genet 27: 1–6. 21106271 10.1016/j.tig.2010.10.004 PMC3020277

[10] Yenerall P, Krupa B, Zhou L (2011) Mechanisms of intron gain and loss in *Drosophila* . BMC Evol Biol 11: 364. 22182367 10.1186/1471-2148-11-364 PMC3296678

[11] Yenerall P, Zhou L (2012) Identifying the mechanisms of intron gain: progress and trends. Biol Direct 7: 29. 22963364 10.1186/1745-6150-7-29 PMC3443670

[12] Belshaw R, Bensasson D (2006) The rise and falls of introns. Heredity 96: 208–213. 16449982 10.1038/sj.hdy.6800791

[13] Fink GR (1987) Pseudogenes in yeast? Cell 49: 5–6. 3549000 10.1016/0092-8674(87)90746-x

[14] Torriani Stefano FF, Stukenbrock Eva H, Brunner Patrick C, McDonald Bruce A, Croll D (2011) Evidence for extensive recent intron transposition in closely related fungi. Curr Biol 21: 2017–2022. 22100062 10.1016/j.cub.2011.10.041

[15] Croll D, McDonald BA (2012) Intron gains and losses in the evolution of *Fusarium* and *Cryptococcus* fungi. Genome Biol Evol 4: 1148–1161. 23054310 10.1093/gbe/evs091 PMC3514964

[16] Coulombe-Huntington J, Majewski J (2007) Characterization of intron loss events in mammals. Genome Res 17: 23–32. 17108319 10.1101/gr.5703406 PMC1716263

[17] Roy SW, Gilbert W (2005) The pattern of intron loss. Proc Natl Acad Sci USA 102: 713–718. 15642949 10.1073/pnas.0408274102 PMC545554

[18] Cohen NE, Shen R, Carmel L (2012) The role of reverse transcriptase in intron gain and loss mechanisms. Mol Biol Evol 29: 179–186. 21804076 10.1093/molbev/msr192

[19] Sharpton TJ, Neafsey DE, Galagan JE, Taylor JW (2008) Mechanisms of intron gain and loss in *Cryptococcus* . Genome Biol 9: R24. 18234113 10.1186/gb-2008-9-1-r24 PMC2395259

[20] Nielsen CB, Friedman B, Birren B, Burge CB, Galagan JE (2004) Patterns of intron gain and loss in fungi. PLoS Biol 2: e422. 15562318 10.1371/journal.pbio.0020422 PMC532390

[21] Lin H, Zhu W, Silva J, Gu X, Buell CR (2006) Intron gain and loss in segmentally duplicated genes in rice. Genome Biol 7: R41. 16719932 10.1186/gb-2006-7-5-r41 PMC1779517

[22] Zhang LY, Yang YF, Niu DK (2010) Evaluation of models of the mechanisms underlying intron loss and gain in *Aspergillus* fungi. J Mol Evol 71: 364–373. 20862581 10.1007/s00239-010-9391-6

[23] Fawcett JA, Rouzé P, Van de Peer Y (2012) Higher intron loss rate in *Arabidopsis thaliana* than *A. lyrata* is consistent with stronger selection for a smaller genome. Mol Biol Evol 29: 849–859. 21998273 10.1093/molbev/msr254

[24] Coulombe-Huntington J, Majewski J (2007) Intron loss and gain in *Drosophila* . Mol Biol Evol 24: 2842–2850. 17965454 10.1093/molbev/msm235

[25] Knowles DG, McLysaght A (2006) High rate of recent intron gain and loss in simultaneously duplicated *Arabidopsis* genes. Mol Biol Evol 23: 1548–1557. 16720694 10.1093/molbev/msl017

[26] Farlow A, Meduri E, Dolezal M, Hua L, Schlotterer C (2010) Nonsense-mediated decay enables intron gain in *Drosophila* . PLoS Genet 6: e1000819. 20107520 10.1371/journal.pgen.1000819 PMC2809761

[27] Feiber AL, Rangarajan J, Vaughn JC (2002) The evolution of single-copy *Drosophila* nuclear *4f-rnp* genes: Spliceosomal intron losses create polymorphic alleles. J Mol Evol 55: 401–413. 12355261 10.1007/s00239-002-2336-y

[28] Niu D-K, Hou W-R, Li S-W (2005) mRNA-mediated intron losses: evidence from extraordinarily large exons. Mol Biol Evol 22: 1475–1481. 15788745 10.1093/molbev/msi138

[29] Cho S, Jin S-W, Cohen A, Ellis RE (2004) A phylogeny of *Caenorhabditis* reveals frequent loss of introns during nematode evolution. Genome Res 14: 1207–1220. 15231741 10.1101/gr.2639304 PMC442136

[30] Roy SW, Hartl DL (2006) Very little intron loss/gain in *Plasmodium*: Intron loss/gain mutation rates and intron number. Genome Res 16: 750–756. 16702411 10.1101/gr.4845406 PMC1473185

[31] Loh Y-H, Brenner S, Venkatesh B (2008) Investigation of loss and gain of introns in the compact genomes of Pufferfishes (Fugu and *Tetraodon*). Mol Biol Evol 25: 526–535. 18089580 10.1093/molbev/msm278

[32] Zhu T, Niu DK (2013) Frequency of intron loss correlates with processed pseudogene abundance: a novel strategy to test the reverse transcriptase model of intron loss. BMC Biol 11: 23. 23497167 10.1186/1741-7007-11-23 PMC3652778

[33] Roy SW, Irimia M (2009) Mystery of intron gain: new data and new models. Trends Genet 25: 67–73. 19070397 10.1016/j.tig.2008.11.004

[34] Li W, Tucker AE, Sung W, Thomas WK, Lynch M (2009) Extensive, recent intron gains in *Daphnia* populations. Science 326: 1260–1262. 19965475 10.1126/science.1179302 PMC3878872

[35] Denoeud F, Henriet S, Mungpakdee S, Aury J-M, Da Silva C, et al (2010) Plasticity of animal genome architecture unmasked by rapid evolution of a pelagic tunicate. Science 330: 1381–1385. 21097902 10.1126/science.1194167 PMC3760481

[36] van der Burgt A, Severing E, de Wit Pierre JGM, Collemare J (2012) Birth of new spliceosomal introns in fungi by multiplication of introner-like elements. Curr Biol 22: 1260–1265. 22658596 10.1016/j.cub.2012.05.011

[37] Worden AZ, Lee JH, Mock T, Rouze P, Simmons MP, et al (2009) Green evolution and dynamic adaptations revealed by genomes of the marine picoeukaryotes *micromonas* . Science 324: 268–272. 19359590 10.1126/science.1167222

[38] Gao X, Lynch M (2009) Ubiquitous internal gene duplication and intron creation in eukaryotes. Proc Natl Acad Sci USA 49: 20818–20823. 10.1073/pnas.0911093106 PMC2791625 19926850

[39] Hankeln T, Friedl H, Ebersberger I, Martin J, Schmidt ER (1997) A variable intron distribution in globin genes of *Chironomus*: evidence for recent intron gain. Gene 205: 151–160. 9461389 10.1016/s0378-1119(97)00518-0

[40] Irimia M, Rukov JL, Penny D, Vinther J, Garcia-Fernandez J, et al (2008) Origin of introns by ‘intronization’ of exonic sequences. Trends Genet 24: 378–381. 18597887 10.1016/j.tig.2008.05.007

[41] Roy SW (2009) Intronization, de-intronization and intron sliding are rare in *Cryptococcus* . BMC Evol Biol 9: 192. 19664208 10.1186/1471-2148-9-192 PMC2740785

[42] Catania F, Lynch M (2008) Where do introns come from? PLoS Biol 6: e283. 19067485 10.1371/journal.pbio.0060283 PMC2586383

[43] Zhu ZL, Zhang Y, Long MY (2009) Extensive structural renovation of retrogenes in the evolution of the *Populus* genome. Plant Physiol 151: 1943–1951. 19789289 10.1104/pp.109.142984 PMC2785971

[44] Szczesniak MW, Ciomborowska J, Nowak W, Rogozin IB, Makalowska I (2011) Primate and rodent specific intron gains and the origin of retrogenes with splice variants. Mol Biol Evol 28: 33–37. 20889727 10.1093/molbev/msq260 PMC3002245

[45] Wood V, Gwilliam R, Rajandream MA, Lyne M, Lyne R, et al (2002) The genome sequence of *Schizosaccharomyces pombe* . Nature 415: 871–880. 11859360 10.1038/nature724

[46] Roy SW, Gilbert W (2005) Rates of intron loss and gain: Implications for early eukaryotic evolution. Proc Natl Acad Sci USA 102: 5773–5778. 15827119 10.1073/pnas.0500383102 PMC556292

[47] Rhind N, Chen Z, Yassour M, Thompson DA, Haas BJ, et al (2011) Comparative functional genomics of the fission yeasts. Science 332: 930–936. 21511999 10.1126/science.1203357 PMC3131103

[48] Galagan JE, Henn MR, Ma LJ, Cuomo CA, Birren B (2005) Genomics of the fungal kingdom: Insights into eukaryotic biology. Genome Res 15: 1620–1631. 16339359 10.1101/gr.3767105

[49] Ng M-P, Vergara I, Frech C, Chen Q, Zeng X, et al (2009) OrthoClusterDB: an online platform for synteny blocks. BMC Bioinformatics 10: 192. 19549318 10.1186/1471-2105-10-192 PMC2711082

[50] Edgar R (2004) MUSCLE: a multiple sequence alignment method with reduced time and space complexity. BMC Bioinformatics 5: 113. 15318951 10.1186/1471-2105-5-113 PMC517706

[51] Felsenstein J (1989) PHYLIP - Phylogeny Inference Package (Version 3.2). Cladistics 5: 164–166.

[52] Kent WJ (2002) BLAT–the BLAST-like alignment tool. Genome Res 12: 656–664. 11932250 10.1101/gr.229202 PMC187518

[53] Drabenstot SD, Kupfer DM, White JD, Dyer DW, Roe BA, et al (2003) FELINES: a utility for extracting and examining EST-defined introns and exons. Nucleic Acids Res 31: e141. 14602934 10.1093/nar/gng141 PMC275578

[54] Crooks GE, Hon G, Chandonia JM, Brenner SE (2004) WebLogo: a sequence logo generator. Genome Res 14: 1188–1190. 15173120 10.1101/gr.849004 PMC419797

[55] Cartegni L, Chew SL, Krainer AR (2002) Listening to silence and understanding nonsense: exonic mutations that affect splicing. Nat Rev Genet 3: 285–298. 11967553 10.1038/nrg775

[56] Maere S, Heymans K, Kuiper M (2005) BiNGO: a Cytoscape plugin to assess overrepresentation of Gene Ontology categories in Biological Networks. Bioinformatics 21: 3448–3449. 15972284 10.1093/bioinformatics/bti551

