# Supplementary material for: Mechanisms of Intron Loss and Gain in the Fission Yeast Schizosaccharomyces
Source: PLoS One. 2013 Apr 17;8(4):e61683. doi: 10.1371/journal.pone.0061683 (PMC3629103; doi:10.1371/journal.pone.0061683)
Supplement: Table S4 — Intron-lost genes and intron-gained genes in fission yeasts have higher expression levels. (DOC) [file pone.0061683.s004.doc]

Table S4. Intron-lost genes and intron-gained genes in fission yeast have higher expression levels

|  | ***n*** | **median** | ***P*-value^c^** |
| --- | --- | --- | --- |
| **Expression level^a^** |  |  |  |
| IL gene^b^ | 383 | 11.31 | 6 × 10^−6^ |
| IG gene^b^ | 55 | 11.38 | 0.014 |
| Other gene | 3,906 | 11.00 |  |

^a^Expression level data for fission yeast *Schizosaccharomyces pombe* were from and were compiled by Daniel C. Jeffares. The values were obtained by log transformation.

^b^IL gene, intron-lost gene; IG gene, intron-gained gene.

^c^Mann-Whitney *U* test was used to calculate the *P* value using other genes as a control set.

**Reference**

1. Jeffares DC, Penkett CJ, Bahler J (2008) Rapidly regulated genes are intron poor. Trends in Genetics 24: 375-378.
