# Supplementary figures and images for: Mechanisms of Intron Loss and Gain in the Fission Yeast Schizosaccharomyces
Source: PLoS One. 2013 Apr 17;8(4):e61683. doi: 10.1371/journal.pone.0061683 (PMC3629103; doi:10.1371/journal.pone.0061683)

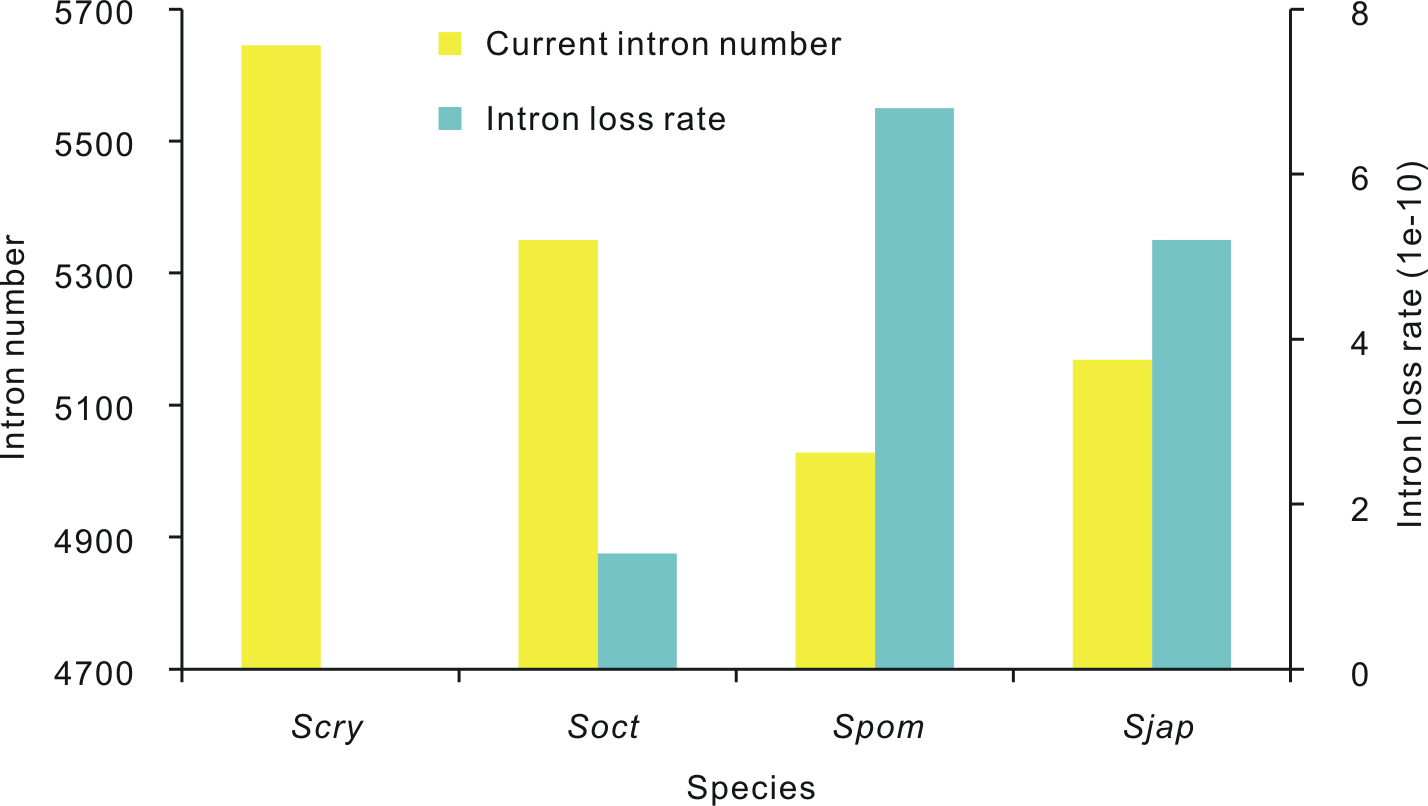

Supplement: Figure S1 — Number of extant introns and intron loss rate are negatively correlated. The intron loss rate and the number of extant introns are negatively correlated. S. cryophilus has the largest number of extant introns and has experienced the lowest intron loss rate, while S. pombe has the fewest introns and had the highest intron loss rate. Species name abbreviations: S. cryophilus (Scry), S. octosporus (Soct), S. pombe (Spom), and S. japonicus (Sjap). (TIF) [file pone.0061683.s005.tif]

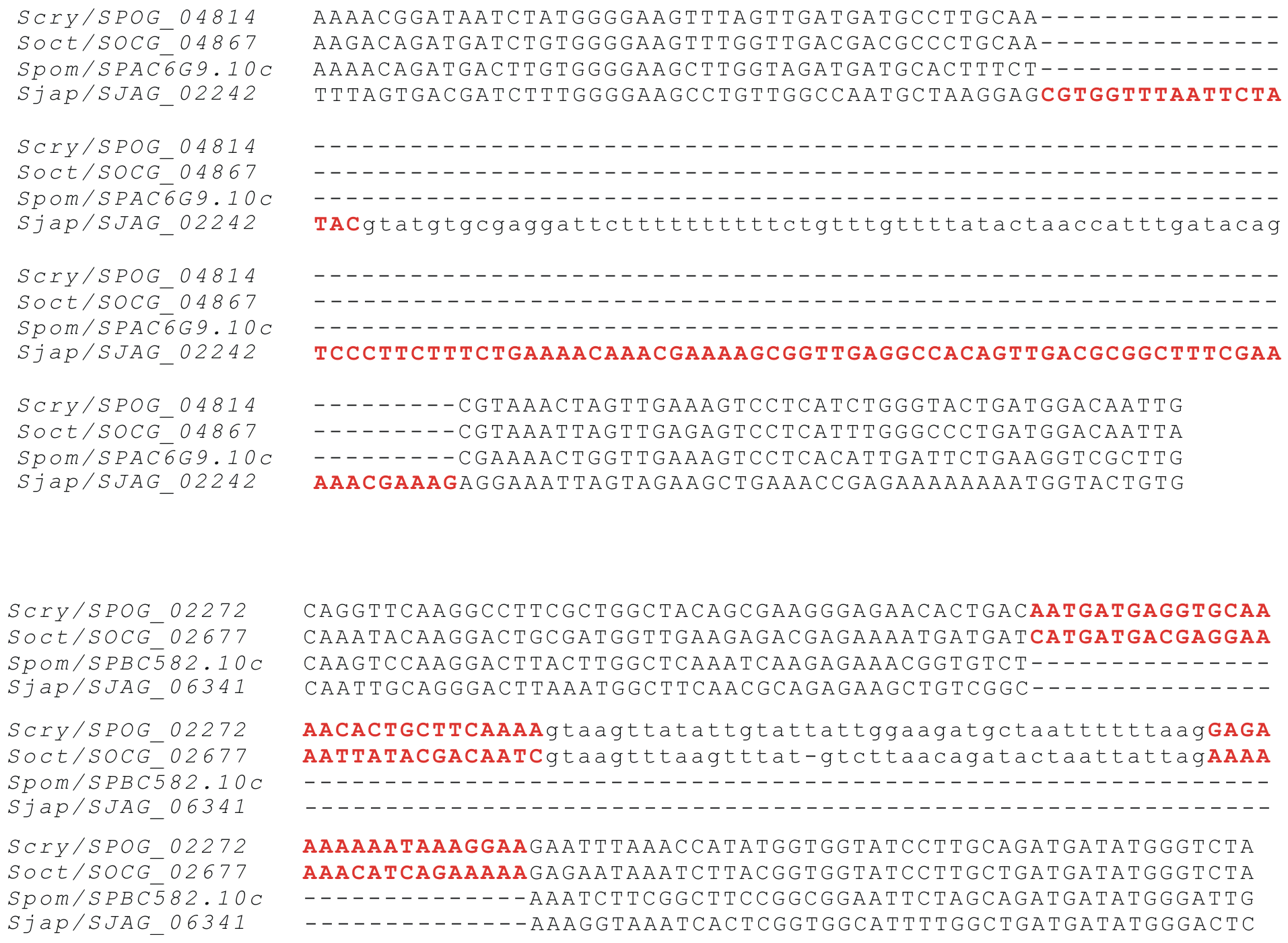

Supplement: Figure S2 — Large indels neighboring unique intron positions in fission yeasts. The alignments of DNA sequences around unique intron regions are shown. Exon sequences are shown in upper case while intron sequences are shown in lower case. Exonic sequence indels accompanying intron loss are marked in red. Species name abbreviations: S. cryophilus (Scry), S. octosporus (Soct), S. pombe (Spom), and S. japonicus (Sjap). (TIF) [file pone.0061683.s006.tif]
