# Supplementary material for: Mechanisms of Intron Loss and Gain in the Fission Yeast Schizosaccharomyces
Source: PLoS One. 2013 Apr 17;8(4):e61683. doi: 10.1371/journal.pone.0061683 (PMC3629103; doi:10.1371/journal.pone.0061683)

**Text S1. The limitation of the Dollop program.** Assume that there were two intron positions as shown below. For both positions, the Dollop program would predict intron gain in *Sjap*. However, intron position A in *Sjap* only had two supported outgroup branches (outgroup *Scom* and node 6, the ancestor of *Scry*, *Soct* and *Spom*) while intron position B in *Sjap* had four supported outgroup branches (node 6 and outgroup *Scom*, *Anig* and *Pbla*). Considering the high rate of intron loss in fission yeast, the data would be more reliable if a minimum number of supported outgroup branches was required. In this study, we required that at least four outgroup branches should be available to support any intron gain events. Species name abbreviations: *S. cryophilus* (*Scry*), *S. octosporus* (*Soct*), *S. pombe* (*Spom*), S. *japonicus* (*Sjap*), *Saitoella complicat* (*Scom*), *Aspergillus niger* (*Anig*), *Nectria haematococca* (*Nhae*), *Sporobolomyces roseus* (*Sros*), *Cryptococcus neoformans* (*Cneo*), *Phycomyces blakesleeanus* (*Pbla*).


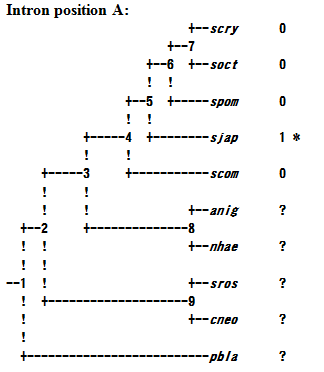

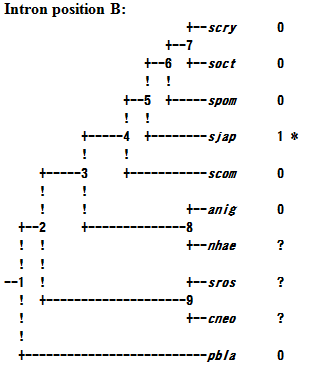

Supplement: Text S1 — The limitation of the Dollop program. (DOC) [file pone.0061683.s007.doc]
